# Nitrogen-Doped Ketjenblack Carbon Supported Co_3_O_4_ Nanoparticles as a Synergistic Electrocatalyst for Oxygen Reduction Reaction

**DOI:** 10.3389/fchem.2019.00766

**Published:** 2019-12-04

**Authors:** Gao Cheng, Guanliang Liu, Peng Liu, Liya Chen, Shengbo Han, Jiaxi Han, Fei Ye, Wei Song, Bang Lan, Ming Sun, Lin Yu

**Affiliations:** ^1^Key Laboratory of Clean Chemistry Technology of Guangdong Regular Higher Education Institutions, School of Chemical Engineering and Light Industry, Guangdong University of Technology, Guangzhou, China; ^2^School of Chemistry and Environment, Jiaying University, Meizhou, China

**Keywords:** Co_3_O_4_, ketjenblack carbon, nitrogen dopant, electronic coupling effect, electrocatalyst, oxygen reduction reaction, zinc-air batteries

## Abstract

Developing a highly active and cost-effective cathode electrocatalyst with strong stability for oxygen reduction reaction (ORR) is extremely necessary. In this work, we reported a facile synthetic path to prepare a hybrid nanostructure formed of nitrogen-doped Ketjenblack carbon (N-KC) supported Co_3_O_4_ nanoparticles (Co_3_O_4_/N-KC), which could be used as a promising and stable electrocatalyst for ORR. Compared with the physical mixture of Co_3_O_4_ and N-KC and pure N-KC samples, the resulting Co_3_O_4_/N-KC nanohybrid afforded remarkably superb ORR activity with a half-wave potential of 0.82 V (vs. reversible hydrogen electrode, RHE) and a limiting current density of 5.70 mA cm^−2^ in KOH solution (0.1 M). Surprisingly, the Co_3_O_4_/N-KC sample possessed a similar electrocatalytic activity but better durability to the 20 wt% Pt/C catalyst. The remarkable ORR activity of the Co_3_O_4_/N-KC nanohybrid was mainly due to the strong coupling effect between Co_3_O_4_ and N-KC, the N species dopant, high electroconductivity, and the large BET surface area. Our work enlightens the exploitation of advanced Co_3_O_4_/carbon hybrid material alternative to the Pt-based electrocatalysts.

## Introduction

With an ever-growing demand in electricity energy supply, electrical energy storage (EES) technology has witnessed booming progress by rapidly exploitative next-generation electrochemical power storage devices, such as supercapacitors (Zhong et al., 2015; Wang S. et al., 2019), Li-ion batteries (Liu et al., 2017; Xia et al., 2017), Zn-ion batteries (Yu et al., 2019), and metal-air batteries (Cheng and Chen, 2012). More recently, zinc-air batteries (ZABs), which generate electricity via the direct redox chemical reaction between Zn and O_2_, have been considered a promising and advanced energy storage device owing to its non-toxicity, low price, reliable safety, as well as a large theoretical energy density of 1,218 Wh kg^−1^ (Fu et al., 2018d; Zhu et al., 2018). Currently, the efficient energy conversion of ZABs is still dramatically hindered by the slow oxygen reduction reaction (ORR) kinetics at cathode, which significantly restricts the quality of ZABs (Guo et al., 2018). To lower the energy barrier of ORR, the rational design and preparation of highly efficient electrocatalysts, is inevitably desirable. Traditionally, the Pt-group metals are the benchmark catalysts for O_2_ electrocatalysis, but their practical utility is greatly plagued by a limited natural shortage, prohibitive prices, and unsatisfying durability (Li et al., 2016; Wang et al., 2017b; Song et al., 2018; Vargas-Ordaz et al., 2019). This issue has led scientists to explore suitable and cost-efficient ORR catalysts with high-efficiency electrochemical activity and stability.

In recent years, lots of inexpensive alternatives have been reported to possess decent ORR activities, including non-precious metals and their alloys (Lee et al., 2018), carbon-based materials (Wu et al., 2016a; Fu et al., 2018b), Fe-N-C (Sun et al., 2018; Wang C. et al., 2019), and transition metal oxides and their derivatives (Meng et al., 2014; Tian et al., 2015; Miura et al., 2016; Jin et al., 2019), etc. Particularly, Co_3_O_4_ has gained growing interest with merits of plentiful resources, eco-friendliness, and mix-valence (Co^2+^ and Co^3+^) (Xiao et al., 2013). However, because of its inherent poor conductivity, the ORR activities of Co_3_O_4_ catalysts are still not satisfactory and underperform the Pt/C catalyst (Yu et al., 2017). Moreover, the Co_3_O_4_ electrocatalysts usually suffer from agglomeration and dissolution during the long-term cycle, giving rise to the insufficient ORR stability (Han et al., 2018). To circumvent these obstacles, carbon-based materials (e.g., graphene, Ketjenblack, and g-C_3_N_4_) with excellent conductivity and corrosion resistance were usually employed as supporters in depositing the nanostructured Co_3_O_4_, which markedly boosts ORR activity and stability (Liang et al., 2011; Liu K. et al., 2016; Li et al., 2017; Wang et al., 2018). Compared to other carbon-based materials, Ketjenblack (KB) has become very attractive by virtue of its favorable physic-chemical properties (e.g., outstanding conductivity, high surface area, and good structural stability), making it an excellent carrier for supporting the Co_3_O_4_ nanocrystallines. For example, the Co_3_O_4_/N-doped KB nanocomposite was successfully obtained through a hydrothermal approach followed by a thermal decomposition process, which achieved a positive half-wave potential [0.79 V vs. reversible hydrogen electrode (RHE)] with surprisingly stability (Liu K. et al., 2016). The Co_3_O_4_/Co-N modified KB nanohybrid was fabricated through a direct pyrolysis method, and displayed a half-wave potential of ~0.80 V vs. RHE with strong durability (Li et al., 2017). Overall, the synthesis of the above reported hybrid ORR catalysts required a long reaction time and complicated procedures, and these catalysts still exhibited dissatisfactory ORR activities. Thereby, the development of a simple strategy for the synthesis of a Co_3_O_4_/KC hybrid electrocatalyst under facile conditions, as well as significantly improved ORR performance, is highly challenging and valuable.

In this context, our facile synthetic strategy realized a hybrid nanostructure of Co_3_O_4_ nanoparticles anchored on a N-doped KC (N-KC) substrate, on which a promising and stable electrocatalytic activity for ORR was accomplished. Due to the strong coupling effect between Co_3_O_4_ and N-KC, the N species dopant, high electroconductivity and large BET surface area, the resultant Co_3_O_4_/N-KC nanohybrid exhibited evidently superior ORR catalytic performance than the Co_3_O_4_ + N-KC and pure N-KC control samples in an alkaline condition. More importantly, such a hybrid Co_3_O_4_/N-KC electrocatalyst showed a comparable ORR performance (half-wave potential and diffusion-limiting current) to that of Pt/C. Additionally, the Co_3_O_4_/N-KC nanohybrid manifested better long-term stability than the Pt/C after 12 h of chronoamperometric measurement. The successful fabrication of the Co_3_O_4_/N-KC hybrid material presented a guideline for the preparation of an effective Pt-free ORR cathode catalyst.

## Experimental Details

### Preparation of N-KC

First, 1.0 g of KC (Ketjenblack, Japan LION, EC-600JD) was slowly added into the concentrated HNO_3_ (50 mL) solution and then heated at 70°C for 6 h. After being cooled to room temperature, KC was washed with deionized water to remove residual surface HNO_3_, and dried at 60°C overnight. Second, 1 g of as-prepared KC and 0.5 g of melamine were well-mixed, sintered at 700°C for 2 h in a N_2_ atmosphere, and cooled naturally, thus the N-KC was finally formed.

### Preparation of Co_3_O_4_/N-KC Nanocomposite and Pure Co_3_O_4_ Nanocubes

Typically, 20.0 mg of Co(CH_3_COO)_2_·4H_2_O was dissolved in 35 ml distilled water to form a clear Co(CH_3_COO)_2_ solution, and then N-KC (70.0 mg) was added into the Co(CH_3_COO)_2_ solution. The obtained slurry was further stirred for at least 20 min. The mixed suspension was rapidly transferred to a Teflon-lined autoclave (50 ml). After hydrothermally reacting at 120°C for 12 h, the resultant precipitate was centrifuged with deionized water and alcohol, and subsequently dried in an oven to yield the Co_3_O_4_/N-KC nanostructure.

Pure Co_3_O_4_ nanocubes were prepared via the same procedures as making a Co_3_O_4_/N-KC sample without adding any N-KC.

### Characterization

The phase purity and crystallinity of products were characterized by X-ray powder diffraction (XRD, Bruker D8 advance) from 2θ = 10–80° (Cu Kα radiation). The shape, microstructure and surface composition of the resulting samples were analyzed by field-emission scanning electron microscopy (FE-SEM, Hitach, SU8220), field-emission transmission electron microscopy (FE-TEM, FEI Talos F200S) and X-ray photoelectron spectroscopy (XPS, Escalab 250Xi). The surface area of Co_3_O_4_/K-NC was calculated from N_2_ adsorption-desorption isotherms at −196°C, attained using an ASAP 2020 surface area analyzer (Micromeritics, USA). The metal contents in the Co_3_O_4_/K-NC were measured on an inductively coupled plasma-optical emission spectrometer (ICP-OES, Agilent 725). The conductivity of the Co_3_O_4_/K-NC and Pt/C samples was measured using a typical two electrode system (ZAHNER, Germany).

### Electrocatalytic Tests

The electrochemical measurements were performed using a rotating device (GAMRY RDE710) with a three-electrode system, lined with a computer-controlled electrochemical workstation (CHI 760E). The working electrode was a glassy carbon (GC) electrode (5.6 mm in diameter) with the catalyst coating. Typically, 7 mg of the sample was dispersed in the mixed solvent (1.0 ml) containing 950 μl of ethanol and 50 μl of Nafion (5 wt%, DuPont), which was then ultrasonicated for ~60 min. The as-obtained ink (7.0 μl) was loaded on the wording electrode and dried in an oven (60°C) for testing, which afforded a mass loading of ~0.20 mg cm^−2^. A platinum foil (1 × 1 cm^2^) and Ag/AgCl electrode were used as the counter electrode and reference electrode, respectively. Before each measurement, a high-purity N_2_/O_2_ flow was used to saturate the aqueous solution (0.1 M KOH) for at least 30 min. The electrocatalytic performances of the samples were tested using a cyclic voltammogram (CV), linear sweep voltammogram (LSV), and a chronoamperometry method. All the obtained potentials were transformed to the RHE scale according to the following Nernst equation (Equation 1).

(1) $$\begin{array}{l} E_{RHE}=E_{Ag/AgCl}+0.059p\text{H}+E_{Ag/AgCl}^{o} \end{array}$$

The electron transfer number (*n*) and the percentage of peroxide species (${\text{HO}}_{2}^{-}$%) were calculated, separately, through the Equation 2 and Equation 3 as follows:

(2) $$\begin{array}{l} n=4\frac{I_{d}}{I_{d}+\frac{I_{r}}{N}} \end{array}$$

(3) $$\begin{array}{l} HO_{2}^{-}\%=200\;\frac{\frac{I_{r}}{N}}{I_{d}+\frac{I_{r}}{N}} \end{array}$$

where *I*_d_, *I*_r_, and *N* represent the disk current, the ring current and the collection efficiency of the RRDE, respectively.

## Results and Discussion

### Structure and Composition Characterization

**Figure 1:**
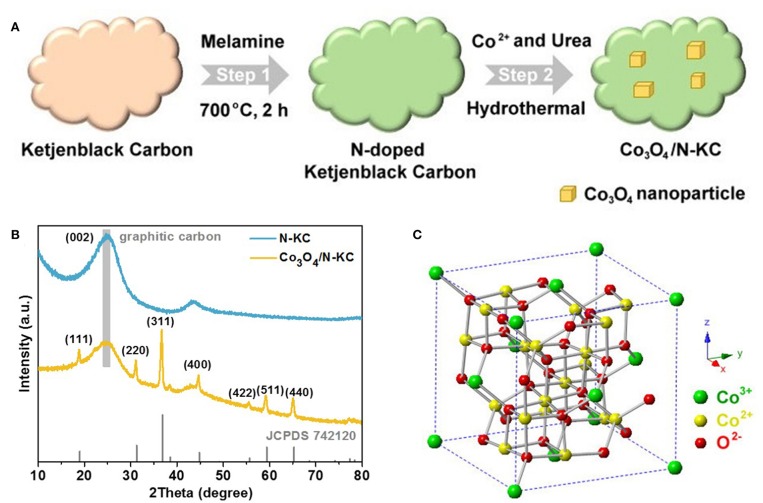
**(A)** Schematic illustration of the synthesis procedures of Co_3_O_4_/N-KC; **(B)** XRD patterns of N-KC and Co_3_O_4_/N-KC; **(C)** Crystalline framework of Co_3_O_4_.

Our synthetic approach of the Co_3_O_4_/N-KC nanocomposite is illustrated in Figure 1A. This method involved two main steps: (i) the synthesis of nitrogen doped KC via calcining the KC with melamine at 700°C under N_2_ flow; and (ii) the growth of Co_3_O_4_ nanoparticles on the N-KC surface through the hydrothermal approach. The crystal structures of N-KC and Co_3_O_4_/N-KC were ascertained by the XRD, as shown in Figure 1B. Obviously, a strong and broad diffraction peak located at ~24.8° can be seen in the XRD patterns of N-KC and Co_3_O_4_/N-KC, which is characteristic of highly graphitic carbon (Ge et al., 2019). For Co_3_O_4_/N-KC, the diffraction peaks at 18.9°, 31.1°, 36.8°, 44.6°, 55.5°, 59.2°, and 65.0° correspond well to the (111), (220), (311), (400), (422), (511), and (440) planes of the spinel Co_3_O_4_ phase (JCPDS: 742120). In the crystalline framework of Co_3_O_4_ (Figure 1C), Co^2+^ cations and Co^3+^ cations occupy the tetrahedral interstices and octahedral interstices, respectively, both of which are coordinated by O^2−^ anions (Xie et al., 2009). Moreover, based on the Scherrer's equation, the particle size of Co_3_O_4_ nanocrystallines on N-KC surface is calculated to be ~26.2 nm.

**Figure 2:**
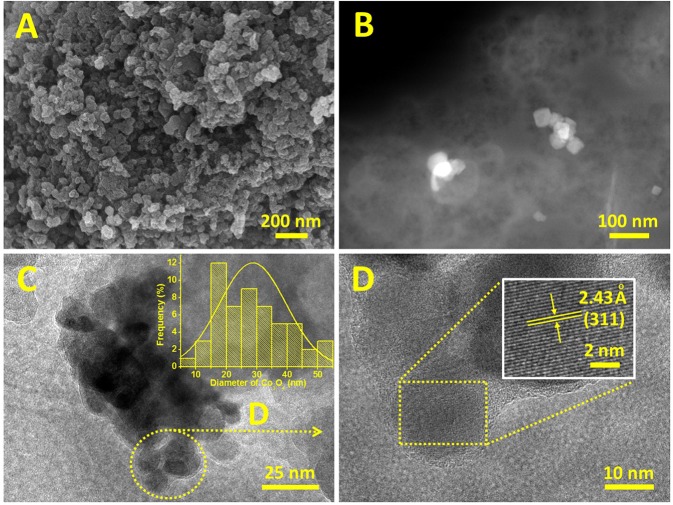
**(A)** FESEM and **(B)** HAADF-STEM images of Co_3_O_4_/N-KC; **(C)** FETEM and size distribution (inset) of Co_3_O_4_ nanoparticles; **(D)** FETEM and HR-FETEM (inset) images of Co_3_O_4_ nanoparticles.

FESEM and FETEM were used to analyze the microstructure of the prepared Co_3_O_4_/N-KC. As observed from the FESEM images (Figure 2A and Figure S1), we can speculate that the Co_3_O_4_ nanocrystallines are well-dispersed throughout the N-KC framework (Liu K. et al., 2016). The high-angle annular dark-field scanning TEM (HAADF-STEM) clearly indicates the presence of Co_3_O_4_ nanoparticles on the N-KC surface (Figure 2B). Moreover, the FETEM image (Figure 2C) further reveals that the Co_3_O_4_ nanocrystallines are densely immobilized on the N-KC surface, exhibiting an average size of ~28.7 nm in the range of 10–50 nm (inset of Figure 2C), which well-approaches the value computed by the XRD result. Shown in Figure 2D is a high magnification of the Co_3_O_4_ nanoparticles from a section of Figure 2C. The corresponding HR-FETEM image (inset of Figure 2D) corroborates that the interlayer spacing is 2.43 Å, which corresponds to the (311) plane of Co_3_O_4_. Such a hybrid nanostructure of Co_3_O_4_/N-KC was characterized via the N_2_ adsorption-desorption measurement. On the basis of the Brunauer-Emmett-Teller (BET) approach, the Co_3_O_4_/N-KC nanohybrid presents an extremely large BET surface area of 813.69 m^2^ g^−1^, as calculated from Figure S2.

**Figure 3:**
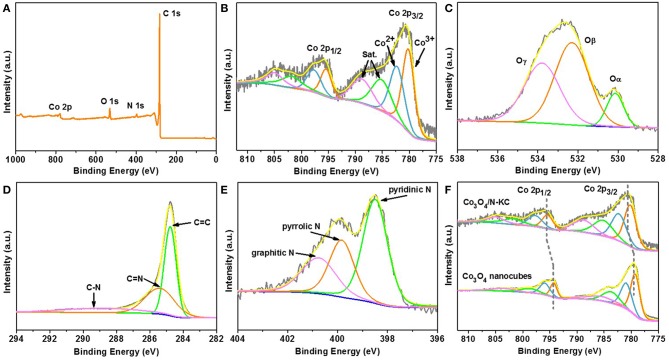
**(A)** XPS full survey spectrum of Co_3_O_4_/N-KC; High-resolution XPS spectra of Co_3_O_4_/N-KC at **(B)** Co 2p, **(C)** O 1s, **(D)** C 1s, and **(E)** N 1s; **(F)** XPS spectra of Co 2p in Co_3_O_4_/N-KC and pure Co_3_O_4_.

The in-depth information on the surface chemical compositions of Co_3_O_4_/N-KC was further studied by the XPS technique. The full survey spectrum in Figure 3A confirms the presence of four kinds of elements (Co, O, N, and C) that can be detected in the Co_3_O_4_/N-KC sample. The existence of N 1s peak demonstrates that the N atoms were successfully doped into the carbon skeleton. In the high-resolution Co 2p spectrum (Figure 3B), two peaks at 780.6 and 795.7 eV are attributed to the Co 2p_3/2_ and Co 2p_1/2_ core levels. In particular, the Co 2p_3/2_ can be broken down into three components of Co^3+^ (780.3 eV), Co^2+^ (782.5 eV), and satellite (785.4 and 789.1 eV), respectively (Ren et al., 2018). Figure 3C presents the O 1s XPS spectrum of Co_3_O_4_/N-KC, which is decomposed into three different types of surface oxygen species including lattice oxygen (O_α_ = 530.2 eV), adsorbed oxygen (O_β_ = 532.3 eV), and chemisorbed water (O_γ_ = 533.8 eV) (Liu K. et al., 2016). As displayed in Figure 3D, the deconvolution of C 1s spectrum exhibits three peaks at 284.7, 285.4, and 288.8 eV, which can be attributable to C=C, C=N, and C-N of the carbon skeleton, respectively (Han et al., 2018). Similarly, the high-resolution spectrum of N 1s can also be fitted to three various forms (Figure 3E), belonging to the pyridinic N (398.5 eV), pyridinic N (399.9 eV), and graphitic N (400.8 eV), respectively (Xie et al., 2015). The Co 2p results of Co_3_O_4_/N-KC were compared to that of a pure Co_3_O_4_ sample, and the detailed structural and morphological results of the Co_3_O_4_ sample are also given in Figure S3. As shown in Figure 3F, it should be noted that the higher binding energy shift of Co 2p_3/2_ and Co 2p_1/2_ peaks can clearly be seen for the Co_3_O_4_/N-KC. This result suggests that there was a strong electronic coupling between Co_3_O_4_ and N-KC, induced by the heterogeneous interface between the Co_3_O_4_ nanoparticles and the N-KC framework (Wu et al., 2016b; Fu et al., 2018c). Taking the above results (XRD, FETEM and XPS) into account, the Co_3_O_4_ nanoparticles immobilized on the N-KC skeleton were successfully prepared through our facile synthetic route.

### ORR Activity and Stability Tests

**Figure 4:**
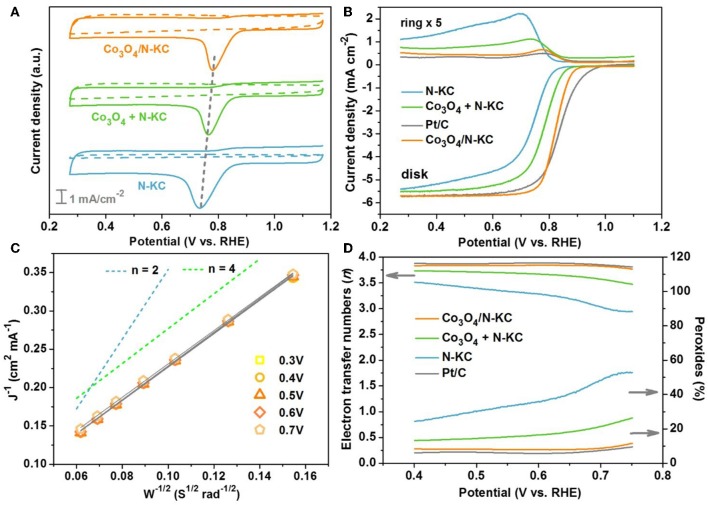
**(A)** CV curves of Co_3_O_4_/N-KC, Co_3_O_4_ + N-KC and N-KC samples in both N_2_-saturated and O_2_-saturated KOH solution (0.1 M); **(B)** LSV curves of Co_3_O_4_/N-KC, Co_3_O_4_ + N-KC, N-KC, and Pt/C at 1,600 rpm; **(C)** K-L plots of Co_3_O_4_/N-KC at various potentials; **(D)** Electron transfer number and percentage of peroxides for Co_3_O_4_/N-KC.

The electrochemical catalytic performances of the Co_3_O_4_/N-KC nanocomposite for ORR was conducted in KOH medium (0.1 M). The cyclic voltammogram (CV) of Co_3_O_4_/N-KC was first carried out and compared with other control samples, including a physical mixture of Co_3_O_4_ nanocubes and N-KC (Co_3_O_4_ + N-KC) and pure N-KC. As depicted in Figure 4A, the CV curves in N_2_-saturated electrolyte on all the samples show no redox peaks. However, in the O_2_-saturated electrolyte, the corresponding CV curves display distinct cathodic peaks, belonging to the ORR electrocatalysis. In particular, the ORR peak of Co_3_O_4_/N-KC is located at 0.78 V, which is the most positive among the three samples, suggesting its extraordinary electrocatalytic activity. The ORR performances of the Co_3_O_4_/N-KC, Co_3_O_4_ + N-KC, and N-KC were further investigated by LSV using a rotating ring-disk electrode (RRDE) at 1,600 rpm. Moreover, the LSV measurement of commercial Pt/C was also evaluated. Figure 4B displays the LSV curves measured by the ring and disk. Based on the data collected from the disk, the Co_3_O_4_/N-KC presents a high onset potential (*E*_onset_) and half-wave potential (*E*_half_) of 0.87 and 0.82 V, respectively, which are much more positive than those of Co_3_O_4_ + N-KC (*E*_onset_ = 0.84 and *E*_half_ = 0.77 V) and N-KC (*E*_onset_ = 0.81 and *E*_half_ = 0.73 V). In addition, the limiting current density at 0.3 V follows the trend of Co_3_O_4_/N-KC (5.70 mA cm^−2^) > Co_3_O_4_ + N-KC (5.51 mA cm^−2^) > N-KC (5.36 mA cm^−2^). Though the *E*_onset_ of Co_3_O_4_/N-KC is lower than that of Pt/C (0.91 V), the values of *E*_half_ and limiting current density afforded by Co_3_O_4_/N-KC are well-close to those of Pt/C (0.83 V and 5.72 mA cm^−2^). Remarkably, the ring current density of Co_3_O_4_/N-KC also highly approaches that of Pt/C, which is obviously lower than those of Co_3_O_4_ + N-KC and N-KC. The results above collectively testify that the prepared Co_3_O_4_/N-KC nanohybrid delivers a remarkable electrocatalytic activity for ORR in alkaline media, which rivals those of the excellent non-noble metal electrocatalysts reported previously (Table S1).

To deeply understand the ORR reaction kinetics for the Co_3_O_4_/N-KC nanohybrid, LSV tests at different rotating speeds (400–2,500 rpm) were further conducted, as illustrated in Figure S4. As expected, the current density value increases with the enhancement of rotation speed since the diffusion distance becomes shortened at an improved rotation rate (Soren et al., 2019). Figure 4C displays the K-L lines of Co_3_O_4_/N-KC based on the rotation-speed-dependent currents under various potentials (0.3–0.7 V), and all of them possess excellent linearity. Significantly, these lines exhibit nearly similar slopes to the green line, implying that Co_3_O_4_/N-KC favors a quasi-four-electron (4e^−^) ORR mechanism (Wu et al., 2018). Figure 4D presents the electron transfer number (*n*) and percentage of peroxide species (${\text{HO}}_{2}^{-}$%) calculated from the ring and disk currents. Within the potential range from 0.40 to 0.75 V, the calculated *n* of Co_3_O_4_/N-KC is ~3.82, which is almost the same with the Pt/C (~3.87), again revealing that the ORR catalyzed by Co_3_O_4_/N-KC was mainly through a quasi-4e^−^ pathway. Furthermore, the determined ${\text{HO}}_{2}^{-}$% of Co_3_O_4_/N-KC (~8.35%) is substantially lower than that of Co_3_O_4_ + N-KC (~17.03%) and N-KC (~36.32%), showing high electrocatalytic efficiency for the ORR.

**Figure 5:**
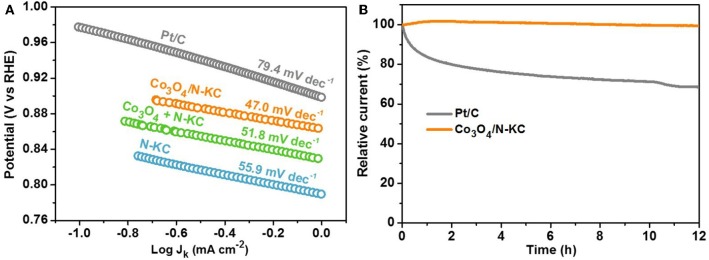
**(A)** Tafel plots of the four samples; **(B)** Chronoamperometry (12 h) of the Co_3_O_4_/N-KC and Pt/C samples maintained at a constant potential of 0.5 V in O_2_-saturated KOH (0.1 M).

The ascendant ORR electrocatalytic activity of Co_3_O_4_/N-KC can be further demonstrated by the Tafel slope (Figure 5A). Amongst all the samples, the Co_3_O_4_/N-KC displays the smallest slope (47.0 mV dec^−1^), which is indicative of its favorable ORR dynamic rate (Liu J. et al., 2016; Wang et al., 2016). Beyond that, chronoamperometry was measured to compare the ORR durability of the Co_3_O_4_/N-KC and Pt/C catalysts (Figure 5B). After testing for a continuous period of 12 h, the Co_3_O_4_/N-KC undergoes a negligible current decrease of 0.51%, while a distinct current fading (31.38%) is achieved for Pt/C. The superb ORR activity as well as outstanding durability of Co_3_O_4_/N-KC signify that the Co_3_O_4_/N-KC nanohybrid can be applied as a promising cathode catalyst in ZABs.

### Origin of ORR Activity of Co_3_O_4_/N-KC

**Figure 6:**
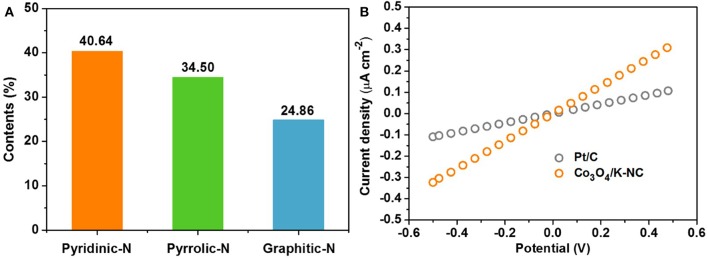
**(A)** The contents of different kinds of N species in Co_3_O_4_/N-KC sample; **(B)** Comparison of I–V curves of the Co_3_O_4_/N-KC and Pt/C samples.

Considering the corresponding physico-chemical characterizations for the Co_3_O_4_/N-KC nanohybrid, its impressive ORR activity could be well-ascribed to the following main aspects: (1) Strong electronic coupling effect. The XPS result, as evidenced through Co 2p spectrum (Figure 3F), suggests that a strong electronic coupling exists in the Co_3_O_4_/N-KC nanohybrid. This strong coupling effect not only guarantees the improved electron transport ability, but also offers highly active electrochemically sites, thereby leading to our hybrid Co_3_O_4_/N-KC catalyst being more active (Liang et al., 2012). (2) Abundant pyridinic and graphitic N species. The contents of pyridinic, pyrrolic and graphitic N species within the Co_3_O_4_/N-KC nanohybrid are estimated to be 40.64, 34.50, and 24.86%, respectively (Figure 6A). It was demonstrated that both the pyridinic and graphitic N dopants in the carbon skeleton could improve the O_2_ capture ability, which further boosted the ORR electrocatalytic process (Xie et al., 2015; Fu et al., 2018a). (3) A highly electroconductive and large BET surface area. The I-V curves of the Co_3_O_4_/N-KC and Pt/C samples measured by two electrode systems are given in Figure 6B. Note that the electrical conductivity of the Co_3_O_4_/N-KC is even higher than the Pt/C, which may be caused by the direct combination of the Co_3_O_4_ nanoparticles and the highly conductive N-KC skeleton. Also, the large BET surface area can offer more sufficient contact between catalyst and molecules/ions. Benefiting from these two distinct advantages, the efficient electrochemical reactions of the Co_3_O_4_/N-KC for ORR can occur (He et al., 2017; Wang et al., 2017a).

## Conclusion

Altogether, a hybrid nanostructure, Co_3_O_4_ nanoparticles supported on a K-NC skeleton, was successfully fabricated through a facile strategy for the application of electrocatalytic oxygen reduction. The strong coupling effect, N species dopant, high electroconductivity, and large BET surface area enabled our Co_3_O_4_/N-KC nanohybrid to be highly efficient, as the ORR cathode electrocatalyst. In contrast to the Co_3_O_4_ + N-KC and pure N-KC samples, the resultant Co_3_O_4_/N-KC nanohybrid exhibited splendid ORR electrocatalytic activity in KOH medium, achieving more positive potential values (*E*_onset_ = 0.87 V and *E*_half_ = 0.82 V) and larger limited current density (5.70 mA cm^−2^). What's more, the Co_3_O_4_/N-KC sample displayed comparable electrocatalytic activity and better stability compared to the Pt/C catalyst.

## Data Availability Statement

The datasets generated for this study are available on request from the corresponding author.

## Author Contributions

GC, GL, PL, and LY designed the project. GC, GL, PL, LC, WS, and BL performed the synthesis and characterization experiments, and data analysis. GL, PL, LC, SH, JH, and FY did the ORR tests and data analysis. MS helped design the ORR tests. GC wrote this manuscript. All authors contributed to editing and discussion of the manuscript.

### Conflict of Interest

The authors declare that the research was conducted in the absence of any commercial or financial relationships that could be construed as a potential conflict of interest.
