# Supplementary material for: Nitrogen-Doped Ketjenblack Carbon Supported Co3O4 Nanoparticles as a Synergistic Electrocatalyst for Oxygen Reduction Reaction
Source: Front Chem. 2019 Dec 4;7:766. doi: 10.3389/fchem.2019.00766 (PMC6904301; doi:10.3389/fchem.2019.00766)
Supplement: Supplementary file 1 [file Table_1.docx]

**Supplementary Material**

**
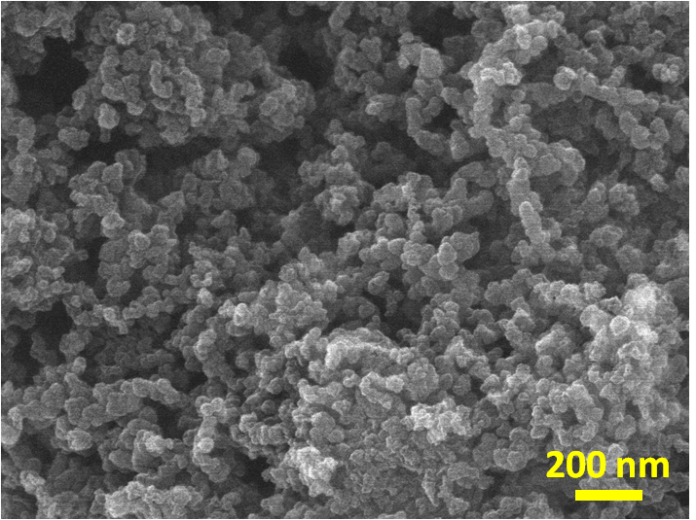
**

**FIGURE S1** FESEM image of the N-KC sample.


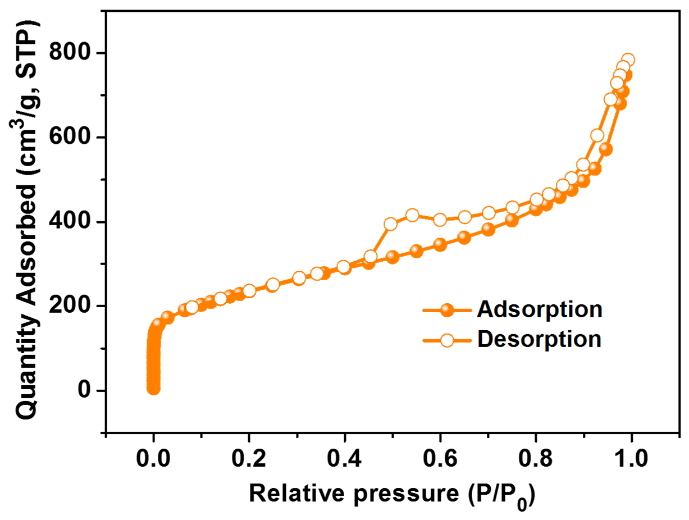


**FIGURE S2** Nitrogen adsorption-desorption isotherm of Co_3_O_4_/N-KC sample.


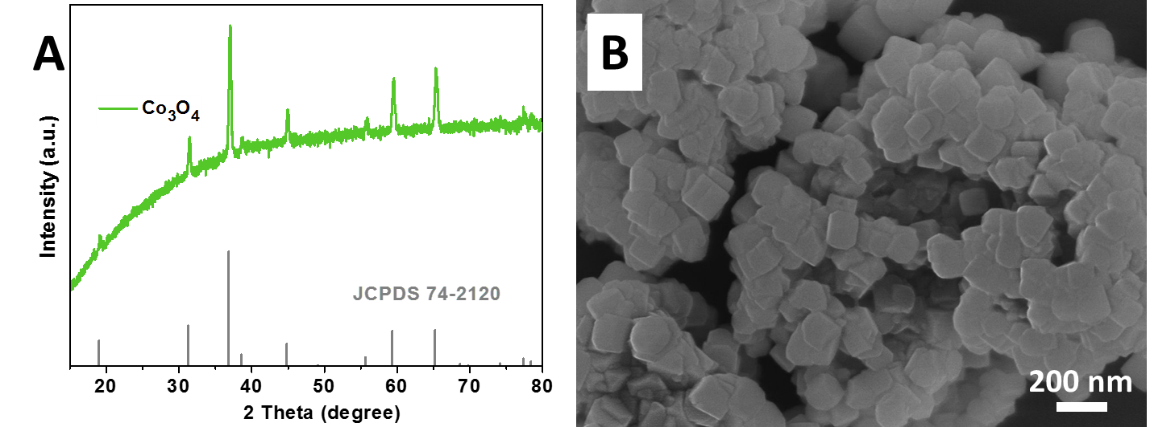


**FIGURE S3** **(A)** XRD pattern and **(B)** FESEM image of the pure Co_3_O_4_ sample.

As shown in the XRD pattern (Figure S3A), all the typical diffraction peaks demonstrate that the Co_3_O_4_ structure with high purity is synthesized (cubic, Fd-3m, a = b = c = 8.084 Å, JCPDS: 742120). Figure S3B indicates the Co_3_O_4_ structure exhibits the shape of nanocube with an average particle size of ~ 85.3 nm.

**FIGURE S4** Co_3_O_4_/N-KC sample in O_2_-saturated KOH (0.1 M) with a scan rate of 5 mV s^−1^ at the different rotation speeds.

**Table S1** Parameters of ORR performance (*E*_onset_ and Limiting current density) for previously reported non-noble metal samples measured in alkaline solution.

| Sample | *E*_onset_ (V) | Limiting current density (mA cm^-2^) | Ref |
| --- | --- | --- | --- |
| Co_3_O_4_/N-KC | 0.87 | 5.70 | This work |
| CoIn_2_S_4_/S-rGO | 0.93 | ~ 5.57 | Fu et al., 2018 |
| Co/CoO/graphene | 0.87 | 4.53 | Guo et al., 2012 |
| Co_3_O_4_-NP/N-rGO | 0.89 | 5.48 | Han, et al., 2018 |
| NiO/MnO_2_@PANI | 0.92 | ~ 5.51 | He et al., 2017 |
| Co_3_O_4_/Co-N-C | 0.83 | 5.10 | Li et al., 2017 |
| N-Carbon/MnO_2_ | 0.88 | 5.57 | Li et al., 2018 |
| Co_3_O_4_/N-rmGO | 0.88 | 4.55 | Liang et al., 2011 |
| CoO_x_/C | 0.86 | 4.52 | Liu et al., 2014 |
| CaMnO_3_/S-300 | 0.92 | 5.52 | Peng. et al., 2018 |

**REFERENCES**

Fu, G., Wang, J., Chen, Y., Liu, Y., Tang, Y., Goodenough, J.B., et al. (2018). Exploring indium-based ternary thiospinel as conceivable high-potential air-cathode for rechargeable Zn-air batteries. *Adv. Energy Mater.* 8:1802263. doi: 10.1002/aenm.201802263

Guo, S., Zhang, S., Wu, L., and Sun, S. (2012). Co/CoO nanoparticles assembled on graphene for electrochemical reduction of oxygen. *Angew. Chem. Int. Ed.* 51, 11770-11773. doi: 10.1002/anie.201206152

Han, X., He, G., He, Y., Zhang, J., Zheng, X., Li, L., et al. (2018). Engineering catalytic active sites on cobalt oxide surface for enhanced oxygen electrocatalysis. *Adv. Energy Mater.* 8:1702222. doi: 10.1002/aenm.201702222

He, J., Wang, M., Wang, W., Miao, R., Zhong, W., Chen, S.Y., et al. (2017). Hierarchical mesoporous NiO/MnO_2_@PANI core-shell microspheres, highly efficient and stable bifunctional electrocatalysts for oxygen evolution and reduction reactions. *ACS Appl. Mater. Interf.* 9, 42676-42687. doi: 10.1021/acsami.7b07383

Li, J., Zhou, Z., Liu, K., Li, F., Peng, Z., Tang, Y., et al. (2017). Co_3_O_4_/Co-N-C modified ketjenblack carbon as an advanced electrocatalyst for Al-air batteries. *J. Power Sources* 343, 30-38. doi: 10.1016/j.jpowsour.2017.01.018

Li, Y., Cao, S., Fan, L., Han, J., Wang, M., and Guo, R. (2018). Hybrid shells of MnO_2_ nanosheets encapsulated by N-doped carbon towards nonprecious oxygen reduction reaction catalysts. *J Colloid Interface Sci* 527, 241-250. doi: 10.1016/j.jcis.2018.05.056

Liang, Y., Li, Y., Wang, H., Zhou, J., Wang, J., Regier, T., et al. (2011). Co_3_O_4_ nanocrystals on graphene as a synergistic catalyst for oxygen reduction reaction. *Nat. Mater.* 10, 780-786. doi: 10.1038/nmat3087

Liu, J., Jiang, L., Tang, Q., Wang, E., Qi, L., Wang, S., et al. (2014). Amide-functionalized carbon supports for cobalt oxide toward oxygen reduction reaction in Zn-air battery. *Appl. Catal. B Environ*. 148-149, 212-220. doi: 10.1016/j.apcatb.2013.10.058

Peng, S., Han, X., Li, L., Chou, S., Ji, D., Huang, H., et al. (2018). Electronic and defective engineering of electrospun CaMnO_3_ nanotubes for enhanced oxygen electrocatalysis in rechargeable Zinc-air batteries. *Adv. Energy Mater.* 8:1800612. doi: 10.1002/aenm.201800612
